# Lipocalin 2 Reduces MET Levels by Inhibiting MEK/ERK Signaling to Inhibit Nasopharyngeal Carcinoma Cell Migration

**DOI:** 10.3390/cancers14225707

**Published:** 2022-11-21

**Authors:** Ju-Pi Li, Chiao-Wen Lin, Cheng-Chen Huang, Yen-Ting Lu, Yu-Ting Ho, Shun-Fa Yang, Chung-Han Hsin

**Affiliations:** 1 School of Medicine, Chung Shan Medical University, Taichung 402, Taiwan; 2 Department of Pediatrics, Chung Shan Medical University Hospital, Taichung 402, Taiwan; 3 Institute of Oral Sciences, Chung Shan Medical University, Taichung 402, Taiwan; 4 Department of Dentistry, Chung Shan Medical University Hospital, Taichung 402, Taiwan; 5 Department of Otolaryngology, Chung Shan Medical University Hospital, Taichung 402, Taiwan; 6 Department of Otolaryngology, St. Martin De Porres Hospital, Chiayi 600, Taiwan; 7 Institute of Medicine, Chung Shan Medical University, Taichung 402, Taiwan; 8 Department of Medical Research, Chung Shan Medical University Hospital, Taichung 402, Taiwan

**Keywords:** NPC, lipocalin 2, metastasis, MET

## Abstract

**Simple Summary:**

Lipocalin-2 (LCN2) participates in multiple cellular processes, such as proliferation, survival, migration, invasion and inflammation. However, the molecular mechanisms involved in the LCN2-mediated cancer metastasis of human nasopharyngeal carcinoma remain poorly understood. Our study found that LCN2 negatively controlled cell invasion and metastasis by increasing the expression level of MET in NPC cells.

**Abstract:**

Nasopharyngeal carcinoma (NPC) is the most common cancer that occurs in the nasopharynx, and it is difficult to detect early. The main cause of death of NPC patients is cancer metastasis. Lipocalin 2 (LCN2) has been shown to be involved in a variety of carcinogenesis processes. Here, we aimed to study the role of LCN2 in NPC cells and determine its underlying mechanism. We found that LCN2 was expressed differently in NPC cell lines, namely HONE-1, NPC-39, and NPC-BM. The down-regulation of LCN2 levels by siRNA targeting LCN2 (siLCN2) increased cell migration and invasion in HONE-1 cells, while the up-regulation of LCN2 levels by transfection with the LCN2 expression plasmid decreased cell migration and invasion in NPC-BM cells. Furthermore, LCN2 levels negatively regulated the phosphorylation of MEK/ERK pathways. The treatment of the specific MEK/ERK inhibitor, U0126, reduced cell migration in HONE-1 cells, whereas the treatment of tBHQ, an ERK activator, enhanced cell migration in NPC-BM cells. Based on the bioinformatics data, there was a moderately negative correlation between LCN2 and MET in metastatic NPC tissues (r = −0.5946, *p* = 0.0022). Indeed, the manipulation of LCN2 levels negatively regulated MET levels in these NPC cells. The treatment of U0126 reduced siLCN2-increased MET levels, while the treatment of tBHQ enhanced LCN2-enhanced MET levels. Interestingly, the down-regulation of MET levels by siMET further decreased siLCN2-enhanced MET levels and cell migration. Therefore, LCN2 inhibits NPC cell migration by reducing MET levels through MEK/ERK signaling.

## 1. Introduction

Nasopharyngeal carcinoma (NPC) is a malignant epithelial carcinoma of the head and neck region that occurs much more frequently in Southeast Asia [1]. Epidemiological data shows that NPC can occur in all age groups and is most commonly diagnosed in adults between the ages of 40 and 50 years old [2]. The incidence and mortality rate of NPC are two- to three-times higher in males compared with females. The main causes of death in NPC patients are local recurrence and distant metastasis. NPC is staged from stage 0 (the earliest stage) to stage IV (the most advanced stage). NPC patients with higher stage numbers generally have a higher chance of it spreading to other parts of the body. Patients with an advanced stage of NPC usually have complications and local metastases and even distant metastases in the bone, lung and liver [3,4]. Three risk factors, including genetic factors, salt-cured foods and viral infections, are highly related to the etiology of NPC [5,6,7]. Since tumors of the nasopharynx are not easily detected, it is difficult to diagnose NPC early. The most urgent task is to find reliable prognostic biomarkers and effective treatments for patients with NPC, especially those with advanced stages of NPC.

Lipocalin-2 (LCN2), also known as neutrophil gelatinase-associated lipocalin, participates in multiple cellular processes, such as proliferation, survival, migration, invasion and inflammation [8]. LCN2 has been found as a biomarker for several diseases and cancers. Previous studies have reported that LCN2 is involved in pathological disorders, especially in inflammation, metabolic disease, neurological disease, and cancers. Recently, an immunohistochemical analysis of NPC tumor specimens demonstrated that LCN2 expression is associated with the survival of NPC patients [9,10]. A high expression of LCN2 has been found to be positively associated with improved 5-year overall survival in NPC patients [10]. Conversely, a recent study showed that a high expression of LCN2 is associated with a poor 5-year relapse-free survival and local relapse-free survival, but not with distant metastasis-free survival, progression-free survival and overall survival between high- and low-LCN2-expression groups in NPC patients [9]. The discrepancy may be the insufficient number of NPC specimens. In any case, it is important to study the molecular mechanism of LCN2 for NPC production.

When normal cells transform into tumor cells, they need special signals to continue to grow, escape apoptosis and induce blood vessel proliferation, and then for the malignant phenotype to further expand to the surrounding areas [11]. The invasion of tumor cells into the surrounding microenvironment is a key step in cancer progression, leading to the formation of metastasis of cancer cells in secondary regions. An ectopic expression of LCN2 has been shown to reduce cellular invasion and metastases in colon cancer [12], in Ras-transformed mouse mammary cells [13] and in NPC cells [10]. A variety of signaling pathways, such as Ras [14], and mitogen-activated protein kinase (MAPK) [15], have been identified to participate in the metastatic process of many cancers as well as NPC [16]. Based on single-cell transcriptomics data, an increased expression of endogenous LCN2 was found in basal respiratory cells and respiratory ciliated cells [17]. However, the role of LCN2 in the carcinogenesis of NPC is still unclear. By manipulating the expression of LCN2 in NPC cell lines, cell growth and migration abilities were investigated. The potential molecular mechanisms in NPC cells were analyzed. In addition, bioinformatics analysis data were used to strengthen our data. We found that LCN2 negatively regulated cell migration by reducing MET levels by inhibiting MEK/ERK signaling.

## 2. Materials and Methods

### 2.1. Bioinformatics

Microarray data were obtained from the NCBI Gene Expression Omnibus database. For example, the GSE103611 dataset contained gene expressions from 48 samples, including 24 NPC tumor tissues with distant metastasis and 24 NPC tumor tissues without distant metastasis. All relevant datasets are publicly available through open access; therefore, no ethics committee approval was required.

### 2.2. Cells and Cell Culture

Two human NPC cell lines, NPC-39 and NPC-BM, were obtained from Dr. MK Chen, Department of Otolaryngology, Changhua Christian Hospital, Taiwan [18]. Human HONE-1 NPC cell line was obtained from the American Type Culture Collection (Manassas, VA, USA). These cells were free from mycoplasma contamination. All three cell lines were cultured in RPMI-1640 medium (Gibco-BRL, Gaithersburg, MD, USA) supplemented with 10% fetal bovine serum (Hyclone Laboratories, Inc., South Logan, UT, USA) plus 10 μg/mL streptomycin and 10 U/mL penicillin (Invitrogen Life Technologies, Carlsbad, CA, USA) and cultured at 37 °C in a humidified atmosphere of a 5% CO2 incubator.

### 2.3. Small Interfering RNA (siRNA) Transfection

The human siRNAs targeting LCN2 (siLCN2) or MET (siMET) and negative control (NT) were purchased from Applied Biosystems Instruments (Foster City, CA, USA). The siLCN2 and siMET sequences targeted LCN2 (NM_005564) CCUCCGUCCUGUUUAGGAAttUUCCUAAACAGGACGGAGGtg and MET (NM_000245) GCACUAGCAAAGUCCGAGAttUCUCGGACUUUGCUAGUGCct, respectively. To silence the specific genes in HONE-1, siRNAs were transfected into cells using lipofectamine RNAiMAX reagents (Invitrogen Life Technologies) according to manufacturer instructions. Two days after transfection, these cells were used for the following experiments.

### 2.4. DNA Construction, Transient Transfection

Human full-length LCN2 and MET expression vectors were constructed as per our previous study [19]. Briefly, the open reading frame (ORF) of the human LCN2 genes from CaSki cells were amplified by polymerase chain reaction (PCR) using the forward primer 5′-GGATCCATGCCCCTAGGTCTCCTGT-3′ followed by a BamHI site and the reverse primer 5′-CTCGAGCTCAGCCGTCGATACACT-3′ followed by a stop codon and an XhoI site. The ORF of the human MET gene from pT3-EF1a-c-Met (addgene, Plasmid #31784) was amplified by PCR using the forward primer 5′-CCTGGTACCATGAAGGCCCCCGCTGTGCTTGCA-3′ followed by a KpnI site and the reverse primer 5′-GAACTCGAGCTATGATGTCTCCCAGAAGGAGGCT-3′ followed by an XhoI site. Both of the human LCN2 or MET cDNAs were cloned into the pcDNA expression vector. After cloning, the cDNA sequences were verified by DNA sequencing (Tri-I Biotech Inc., Taipei, Taiwan). The pcDNA-LCN2 expression vector, pcDNA-MET expression vector, or pcDNA control vector were transfected into NPC-BM cells using LipofectAMINE 2000 (Invitrogen) according to the manufacturer’s protocols. Two days after transfection, these cells were used for the following experiments.

### 2.5. Cell Growth

Cell growth was assessed by a microculture tetrazolium colorimetric assay. At each respective time point, (3-(4,5-dimethylthiazol-2-yl)-2,5-diphenyltetrazolium bromide) (MTT) solutions were added into the treated cells for 2–4 h, and then the media were removed. The formazan crystals formed by the cells were dissolved using DMSO. The test wavelength at 570 nm and a reference wavelength at 630 nm absorbance was read by a spectrophotometer (Instruments, USA). The measured absorbance was the 630 nm background absorbance subtracted from the 570 nm measurement (OD570 nm-OD630 nm). The daily growth rates were calculated using the absorbance on day 1 as a baseline and measured for 4 days.

### 2.6. Cell Migration and Invasion Assays

Cell migration and invasion assays were performed using a Boyden chamber (pore size, 8 μm) (Neuro Probe, Cabin John, MD, USA) [20]. The Boyden chamber assay was used to study the migration (seeding cells with uncoated filter) and invasion (seeding cells with Matrigel-coated filter) abilities. After transfection, the cells were harvested and seeded in the upper chamber. The migrated and invaded cells were fixed by 100% methanol and stained with 10% Giemsa stain. The number of cells was counted using an Olympus CKX41 microscope (Olympus Corporation, Tokyo, Japan).

### 2.7. Reverse Transcription-Polymerase Chain Reaction (RT-PCR) and Real-Time Quantitative PCR

Total RNAs were extracted using a Total RNA Mini Kit (Geneaid, New Taipei City, Taiwan) and reverse transcribed into complementary DNA (cDNA) using a high-capacity cDNA Reverse Transcription Kit (Applied Biosystems, Foster City, CA, USA). The cDNA synthesis and the PCR amplification assay (RT-PCR) were performed as described by previous studies [21]. Real-time quantitative PCR (qPCR) was performed using an ABI 7500 real-time PCR system (Applied Biosystems, Foster City, CA, USA) as per a previous study [19]. In brief, 20 μL of the reaction mixture was prepared with 10 μL 2× SYBR master mix, 1 μL primers, 2 μL cDNA and 7 μL nuclease-free water. The reaction mixture was initially denatured at 95 °C for 10 min followed by 40 cycles of denaturation at 95 °C for 15 s and annealing and extension at 60 °C for 30 s. For LCN2, the following forward (F) primers and reverse (R) primers were used: F: 5′-TGATCCCAGCCCCACCT-3′, R: 5′-CCAC TTCCCCTGGAATTGGT-3′. For MET, the following were used: F: 5′-ATACGGTCCTATGGCTGGTG-3′, R: 5′-TTGAGAGGTTCTTTCCACCAAGT-3′. The relative mRNA expression was analyzed through the Ct method and was normalized to GAPDH expression.

### 2.8. Western Blot Analysis

The total cell lysates were prepared as previously described in previous studies [22]. Briefly, an equal amount of proteins were fractionated on SDS-polyacrylamide gel electrophoresis and transferred to nitrocellulose membranes. The membranes were blocked using 5% skimmed milk in Tris buffer saline-0.1% Tween20 at room temperature for 1 h. After blocking, the membranes were incubated with various primary antibodies overnight at 4 °C. After washing, the membranes were incubated with the secondary antibody linked with horseradish peroxidase (Sigma-Aldrich, St. Louis, MO, USA) at room temperature for 1 h. After washing, the membranes were developed by an enhanced chemiluminescence kit (Sigma-Aldrich) and visualized by an imaging system. The intensity of the protein bands was measured and quantified was measured via an analysis system (AlphaImager 2000, Alpha Innotech Corporation, and San Leandro, CA, USA). The blots were normalized with their total protein levels. In the study, primary antibodies specific for p38, phosphorylated p38 and β-actin were obtained from BD Biosciences (San Jose, CA, USA). Primary antibodies specific for MET, MEK1/2, ERK1/2, JNK1/2, phosphorylated src, c-raf, MEK1/2, ERK1/2 and JNK1/2 were purchased from Cell Signaling Technology (Danvers, MA, USA). Primary antibodies specific for LCN2 were purchased from R&D Systems (Minneapolis, MN, USA).

### 2.9. Statistical Analysis

Statistically significant differences were calculated using one-way ANOVA followed by Tukey’s test. When two groups were compared, the data were analyzed by using Student’s *t*-test. Significance was set at *p* < 0.05. The presented values are the means standard deviation (SD) of at least three independent experiments.

## 3. Results

### 3.1. NPC Cell Growth Is Not Affected by Differential LCN2 Levels

**Figure 1:**
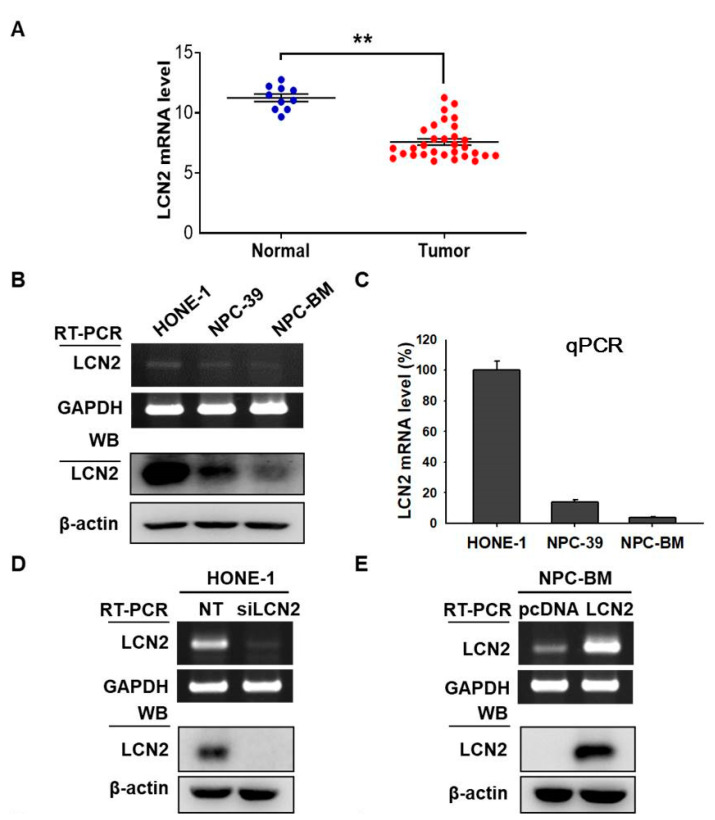

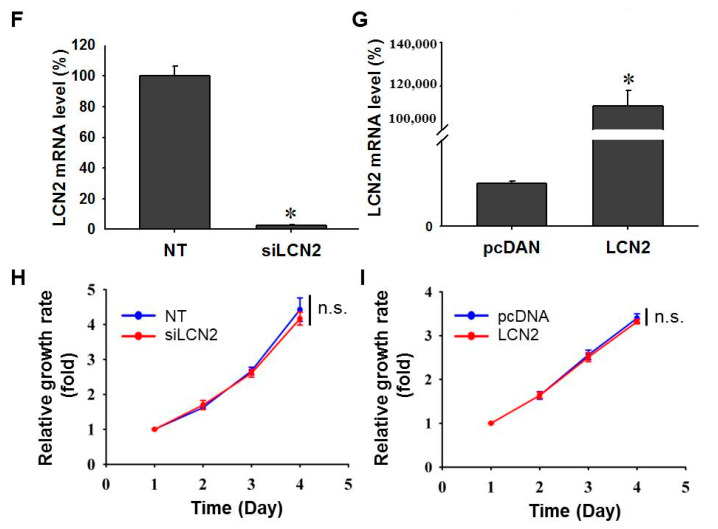
LCN2 did not affect cell growth of human NPC cells. (**A**) Bioinformatics analysis of LCN2 mRNA levels in normal and tumor tissues. ** *p* < 0.05. (**B**) LCN2 mRNA and protein levels in three human NPC cell lines, where HONE-1, NPC-39 and NPC-BM, were determined using RT-PCR and Western blot (WB) analysis. (**C**) LCN2 mRNA levels in three human NPC cell lines were examined using real-time qPCR analysis. (**D**) LCN2 protein and mRNA levels in HONE-1 cells after transfection with siRNA against LCN2 (siLCN2) or control (NT) were examined. (**E**) LCN2 protein and mRNA levels in NPC-BM cells after transfection with control plasmids (pcDNA) or plasmids containing LCN2 (LCN2). (**F**) LCN2 mRNA level of HONE-1 cells after transfection as (**D**) was determined using real-time qPCR analysis. * *p* < 0.05 when compared with control (pcDNA). (**G**) LCN2 mRNA level of NPC-BM cells after transfection as (**E**) was determined using real-time qPCR analysis. (**H**) Cell growth of HONE-1 cells after transfection as (**D**) was determined for 4 days using an MTT assay. (**I**) Cell growth of NPC-BM cells after transfection as (**E**) was determined for 4 days using an MTT assay. n.s.: no significance, when compared with control.

To study the role of LCN2 in NPC progression, we analyzed the dataset (GSE12452) from the Gene Expression Omnibus (GEO) database and found that the expression levels of LCN2 were significantly decreased in the tumor group (*n* = 31) compared with the normal group (*n* = 10) (Figure 1A). In order to explore whether LCN2 was expressed in human NPC cell lines, the levels of LCN2 in three human NPC cell lines, HONE-1, NPC-39 and NPC-BM, were determined using RT-PCR, real-time qPCR and Western blot analysis. Among them, the HONE-1 cell line showed higher LCN2 mRNA and protein levels, while the NPC-BM cell line had lower LCN2 levels (Figure 1B,C). To assess the effect of LCN2 levels on NPC cells, LCN2 expression was down-regulated by the transfection of siRNA targeting LCN2 (siLCN2) in the HONE-1 cells; conversely, LCN2 expression was up-regulated by the transfection of LCN2-expressing plasmids in the NPC-BM cells. As expected, both of the LCN2 mRNA and protein levels were efficiently reduced in the siLCN2-transfected HONE-1 cells compared to those in the HONE-1 cells transfected with control siRNA (Figure 1D). The LCN2 mRNA and protein levels were significantly enhanced in the LCN2-overexpression NPC-BM cells compared to those in the control plasmid-transfected NPC-BM cells (pcDNA) (Figure 1E). The whole Western blot can be found in Figure S1.

The effects of LCN2 levels on cell growth were first examined. As shown in Figure 1F,H, the reduced LCN2 levels in the HONE-1 cells did not interfere with the cell growth rates for up to 4 days, while the enhanced LCN2 levels in the NPC-BM cells also did not interfere with the cell growth rates (Figure 1G,I). These data suggested that different LCN2 levels did not affect the cell growth of the NPC cells.

### 3.2. NPC Cell Migration and Invasion Are Negatively Regulated by LCN2 Levels

**Figure 2:**
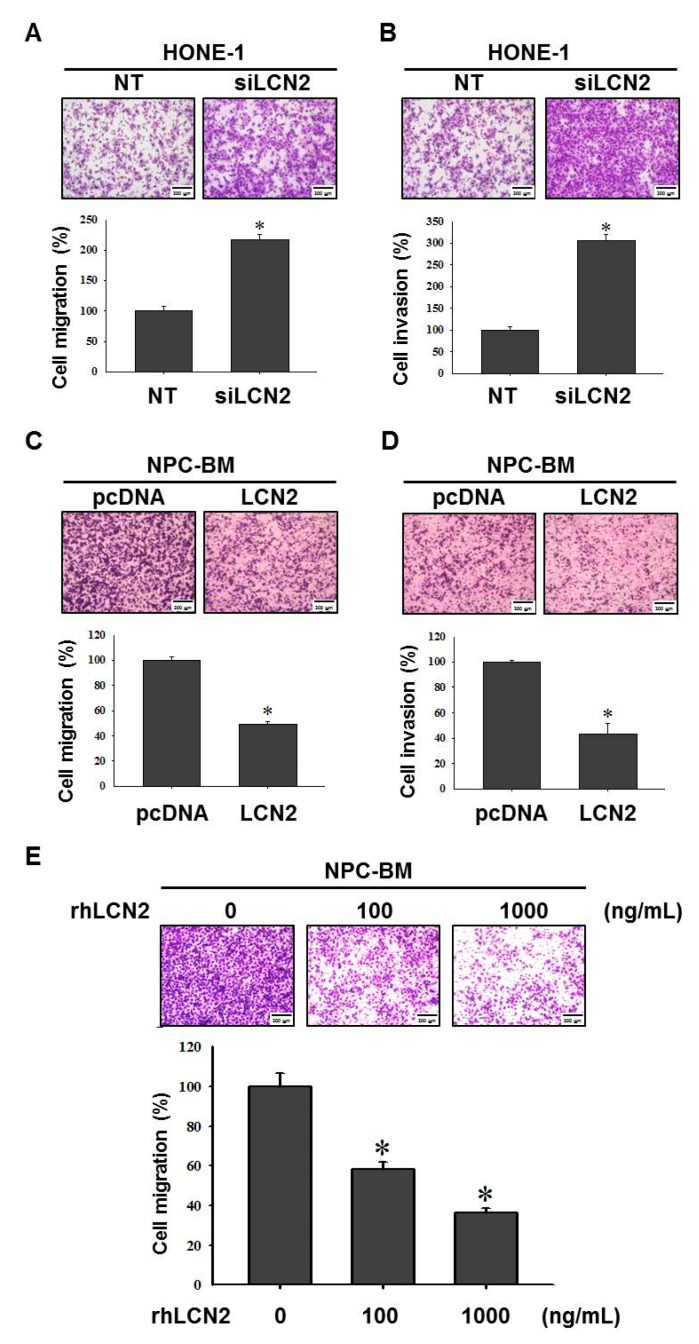
LCN2 negatively regulated cell migration and invasion of human NPC cells. The HONE-1 cells were transfected with siRNA against LCN2 (siLCN2) or control (NT). After siRNA transfection, the HONE-1 cells were examined in terms of cell migration (**A**) and cell invasion (**B**). The NPC-BM cells were transfected with control plasmids (pcDNA) or plasmids containing LCN2 (LCN2). After plasmids transfection, the NPC-BM cells were examined in terms of cell migration (**C**) and cell invasion (**D**). (**E**) NPC-BM cells were treated with recombinant human LCN2 (rhLCN2, 0–1000 ng/mL) and then examined in terms of their cell migration ability. * *p* < 0.05 compared with control.

Next, we explored the effect of LCN2 levels on cell migration and invasion in the NPC cell lines. The knockdown of LCN2 levels significantly enhanced the migration and invasion of the HONE-1 cells (*p* < 0.05) (Figure 2A,B), while the overexpression of LCN2 levels significantly repressed the migration and invasion of the NPC-BM cells (Figure 2C,D). To examine the direct effects of LCN2 protein levels on NPC migration, recombinant human LCN2 proteins (rhLCN2) were used in the NPC-BM cells. As shown in Figure 2E, the treatment of rhLCN2 efficiently suppressed the migration of the NPC-BM cells in a dose-dependent manner. These data demonstrated that the levels of LCN2 inversely correlated with the migration and invasion of the NPC cells.

### 3.3. LCN2 Reduces Cell Migration by Inhibiting the MEK/ERK Signaling

**Figure 3:**
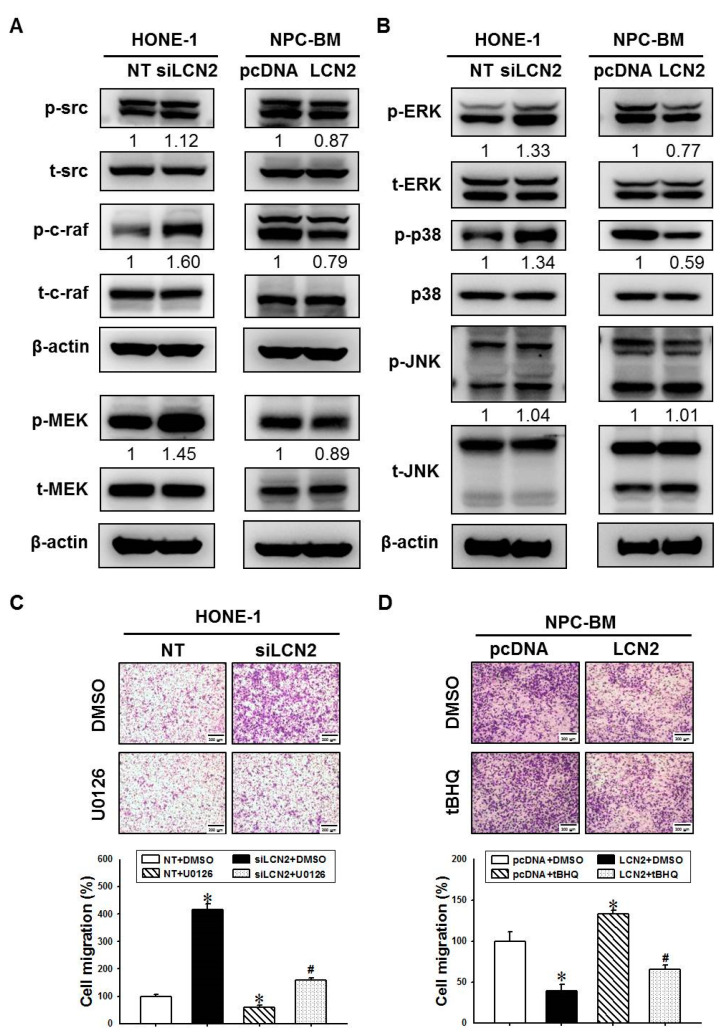
LCN2 suppressed the migration-associated signal cascades to modulate cell migration in human NPC cells. (**A**,**B**) The HONE-1 cells were transfected with siRNA against LCN2 (siLCN2) or control (NT) (left panel). The NPC-BM cells were transfected with control plasmids (pcDNA) or plasmids containing LCN2 (LCN2) (right panel). The migration-associated signal proteins, including their phosphorylation levels, were examined using Western blot analyses. (**C**) The HONE-1 cells that were transfected with siLCN2 or NT and then treated with the MEK/ERK inhibitor, U0126. After treatment, these cells were subjected to cell migration analysis. (**D**) The NPC-BM cells that were transfected with pcDNA or LCN2 plasmids and then treated with the ERK activator, tBHQ. After treatment, these cells were subjected to cell migration analysis. * *p* < 0.05 compared with control. # *p* < 0.05 when compared with HONE-1 cells that were transfected with NT plus U0126, or NPC-BM cells that were transfected with pcDNA plus tph.

Previous studies have shown that mitogen-activated protein kinases (MAPKs), including ERK, p38 and Jun N-terminal kinase (JNK), play key roles in cell migration [23]. To explore the role of LCN2 in the molecular mechanisms of cell migration, we further analyzed these signaling molecules associated with cell migration. As shown in Figure 3A,B, the downregulation of LCN2 in the HONE-1 cells increased the phosphorylation levels of several signaling proteins, such as phospho-src (p-src), phospho-c-raf (p-c-raf), phospho-MEK (p-MEK), phospho-ERK (p-ERK) and phospho-p38 (p-p38) but not phospho-JNK (p-JNK), while the overexpression of LCN2 in the NPC-BM cells decreased the phosphorylation levels of these signaling proteins, including p-src, p-c-raf, p-MEK, p-ERK and p-p38. These data suggested that LCN2 may negatively regulate these motility-associated signal pathways, such the MEK/ERK signaling pathway, leading to a reduction in cell migration and invasion in the NPC cells.

U0126 is a highly selective inhibitor of MEK [24] and is widely used as an inhibitor for the Ras/Raf/MEK/ERK pathway. To determine whether the LCN2-suppressed MEK/ERK signal pathway participates in the regulation of cell migration, HONE-1 cells with or without LCN2 knockdown were co-treated with U0126. The treatment of U0126 significantly reduced cell migration in the HONE-1 cells (Figure 3C, left panel). Additionally, the treatment of U0126 also significantly decreased siLCN2-induced cell migration in the HONE-1 cells (Figure 3C, right panel). *Tert*-butylhydroquinone (tBHQ) has been identified as an ERK activator [25]. The treatment of tBHQ enhanced cell migration in the NPC-BM cells with or without LCN2 overexpression (Figure 3D). These data suggested that LCN2 downregulated cell migration by inhibiting the MEK/ERK signaling pathway.

### 3.4. LCN2 Negatively Regulated MET Levels by Inhibiting the MEK/ERK Signaling Pathway

**Figure 4:**
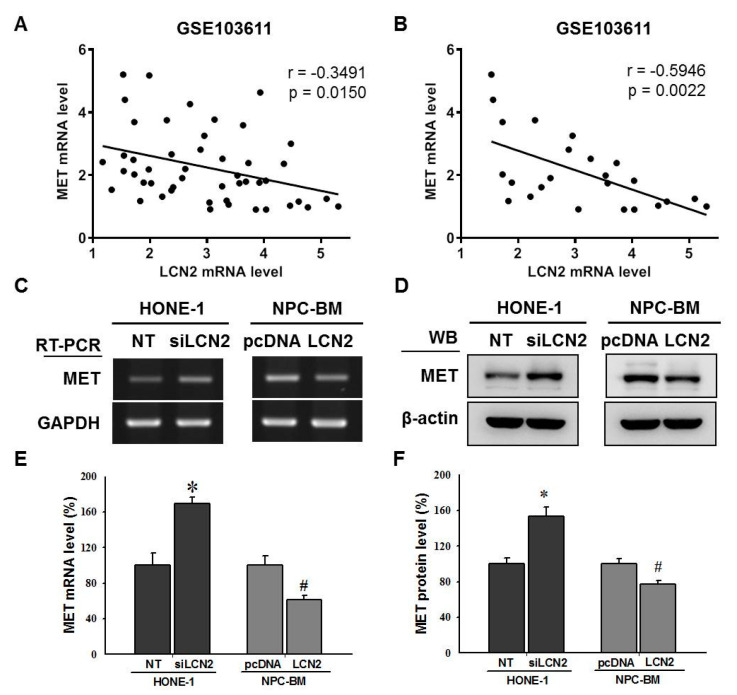

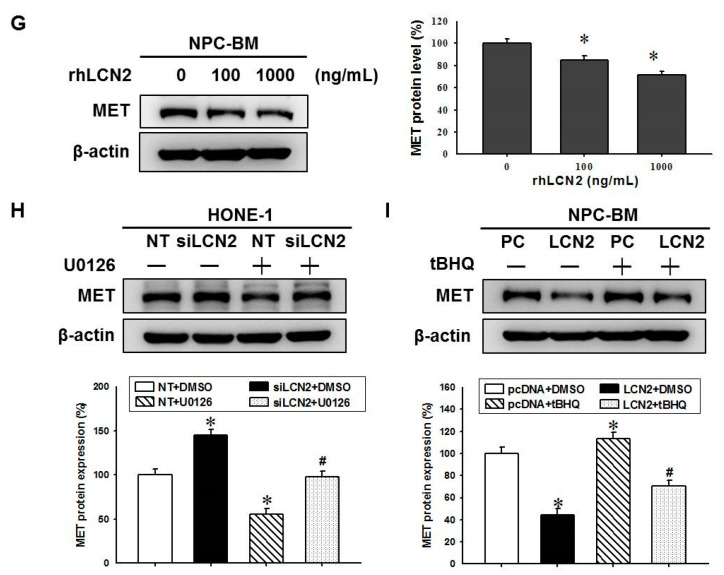
LCN2 down-regulated MET levels through the MEK/ERK cascade in human NPC cells. (**A**) The Pearson correlation between LCN2 and MET mRNA levels in samples from metastatic and non-metastatic NPC patients (*n* = 48) were examined based on GSE103611. (**B**) The Pearson correlation between LCN2 and MET mRNA levels in samples from metastatic NPC patients (*n* = 24) were examined based on GSE103611. (**C**,**D**) The HONE-1 cells were transfected with siRNA against LCN2 (siLCN2) or control (NT) (left panel). The NPC-BM cells were transfected with control plasmids (pcDNA) or plasmids containing LCN2 (LCN2) (right panel). MET mRNA and protein levels were determined using RT-PCR and Western blot (WB) analysis. (**E**) MET mRNA level after transfection as (**C**) was using real-time qPCR analysis. (**F**) Quantitative analysis of MET protein levels in (**D**). (**G**) NPC-BM cells were treated with recombinant human LCN2 (rhLCN2, 0–1000 ng/mL) and then examined in terms of MET protein levels. * *p* < 0.05 when compared with control. (**H**) The HONE-1 cells that were transfected with siLCN2 or NT and then treated with the MEK/ERK inhibitor, U0126. Then, MET protein levels in these cells were examined by Western blot analysis. (**I**) The NPC-BM cells that were transfected with pcDNA or LCN2 plasmids and then treated with the ERK activator, tBHQ. MET protein levels in these cells were examined by Western blot analysis. * *p* < 0.05 compared with control. # *p* < 0.05 when compared with HONE-1 cells that were transfected with NT plus U0126 or NPC-BM cells that were transfected with pcDNA plus tBHQ.

Our recent study has shown that LCN2 suppresses MET expression to inhibit osteosarcoma cell metastasis [26]. We investigated the relationship between LCN2 and MET mRNA expression in samples from metastatic and non-metastatic NPC patients based on the microarray data (GSE103611) obtained from the GEO databases. We found a moderately negative correlation between LCN2 and MET mRNA in these NPC tissues (*r* = −0.3491, *p* = 0.015) (Figure 4A). Further examination of the samples from metastatic NPC patients revealed a more significant negative correlation between LCN2 and MET mRNA (*r* = −0.5946, *p* = 0.0022) (Figure 4B). Indeed, the MET mRNA and protein levels in the siLCN2-transfected HONE-1 cells were significantly enhanced (Figure 4C–F), while the MET mRNA and protein levels in the LCN2-overexpression NPC-BM cells were reduced (Figure 4C–F). The treatment of rhLCN2 proteins also suppressed the expression of MET proteins in the NPC-BM cells in a dose-dependent manner (Figure 4G). Interestingly, the MET levels were repressed by the specific MEK/ERK inhibitor, U0126, in the HONE-1 cells transfected, or not, with siLCN2 (Figure 4H), while the MET levels were slightly enhanced by the ERK activator, tBHQ, in the NPC-BM cells transfected, or not, with LCN2 (Figure 4I). These data demonstrated that LCN2 negatively regulated MET levels through the MEK/ERK signaling pathway.

### 3.5. LCN2 Decreased Cell Migration by Reduced MET Expression

**Figure 5:**
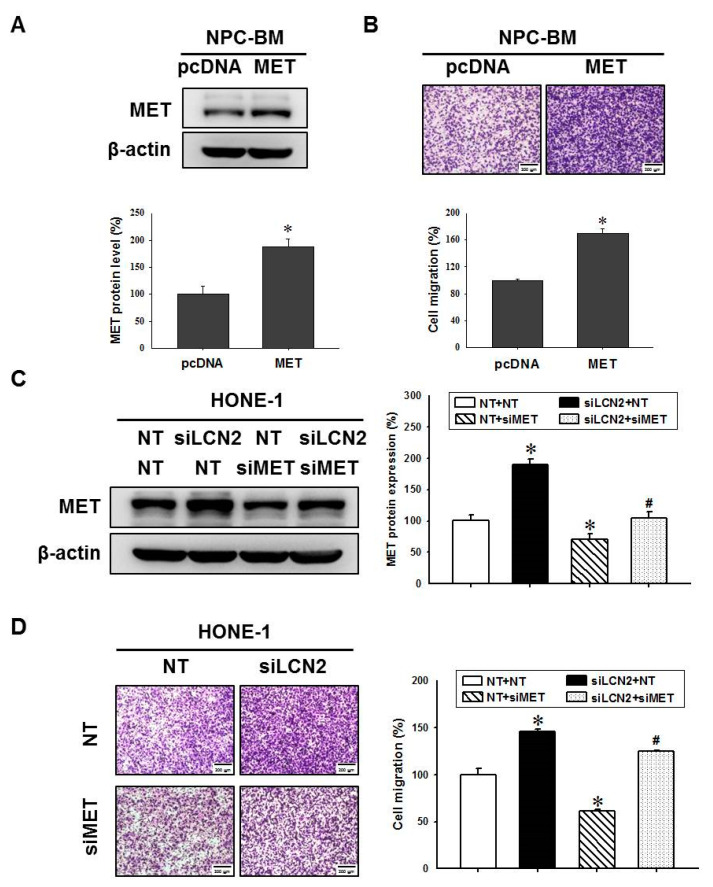
LCN2 decreased cell migration by reducing MET levels in human NPC cells. NPC-BM cells were transfected with control plasmids (pcDNA) or plasmids containing MET (MET) pcDNA and then subjected to Western blot analysis (**A**) or cell migration analysis (**B**). * *p* < 0.05 compared with control. HONE-1 cells were transfected with siRNA against LCN2 (siLCN2), MET (siMET) or control (NT) and then subjected to Western blot analysis (**C**) or cell migration analysis (**D**). * *p* < 0.05 compared with control. # *p* < 0.05 when compared with HONE-1 cells that were transfected with siMET.

The level of MET in NPC tissues is directly and positively correlated with the clinical stage of NPC patients [27]. The downregulation of MET levels efficiently suppresses invasion and migration of NPC cells [28,29]. Thus, we further examined whether LCN2 negatively regulates cell migration by repressing MET levels. The MET protein was overexpressed in the NPC-BM cells (Figure 5A). As expected, the overexpressed MET levels could enhance cell migration in the NPC-BM cells (Figure 5B) similarly to previous studies. Conversely, the HONE-1 cells with reduced MET levels by a specific siRNA-targeting MET (siMET) exhibited a down-regulation of cell migration (Figure 5C,D). It is worth noting that the reduced MET levels also decreased siLCN2-induced cell migration (Figure 5C,D). The data suggested that LCN2 negatively regulated NPC cell migration by reducing MET levels.

## 4. Discussion

Local recurrence and distant metastasis are the main causes of death in patients with NPC. In this study, we demonstrated that LCN2 suppressed NPC cell migration by reducing MET levels through the MEK/ERK signaling pathway. In the NPC cells, LCN2 repressed the MET expression levels, leading to a reduction in the metastasis ability.

Here, we found that the manipulation of LCN2 expression levels did not affect cell growth in the HONE-1 and NPC-BM cell lines; however, Guo et al. showed that the overexpression of LCN2 inhibits cell proliferation in C666 and HNE-3 cell lines [10]. The possible reasons include: (1) The NPC-BM cell line is the first primary NPC cell line derived from a distant metastatic site, i.e., the bone marrow metastatic lesion [30]. It would help to study the real the progression of NPC. (2) Endogenous LCN2 expression of C666 and HNE-3 cells is higher than that of HONE-1 cells [10]. Ectopic overexpression of LCN2 in C666 and HNE-3 cells may induce other growth signals. (3) The C666-1 cell line is EBV-positive and shows a hypermutant phenotype. Therefore, the discrepancy in cell growth may be affected by the different cellular backgrounds. Regardless of the different cell growth results, our study and the study of Guo et al. [10] have shown that LCN2 negatively regulates the invasion and metastasis ability of NPC cells.

LCN2 has been show to participate in the initiation, progression and metastasis of various cancer types [31,32]. However, the role of LCN2 in the different processes of cancers is controversial [33,34]. In this study, we demonstrated that LCN2 negatively regulated the invasion and metastasis of NPC cells using the manipulation of LCN2 in a higher LCN2 expression of NPC-BM cells and lower LCN2 expression of HONE-1 cells. A recent study showed that a high expression of LCN2 was shown in NPC patients with a better prognosis using an immunohistochemical analysis of LCN2 tumor specimens [10]. Furthermore, LCN2 suppresses tumor metastasis in osteosarcoma and oral squamous cell carcinoma [35]. Using computational and immunohistochemistry examinations, LCN2 expression has been shown to be a suppressor of invasion and angiogenesis in six cancer types, such as bladder, colorectal, liver, lung, ovarian and pancreatic when compared to matched primary lesions [36]. On the contrary, LCN2 is positively associated with metastasis in breast cancer [37], anaplastic thyroid carcinoma cells [38] and prostate cancer [39]. LCN2 has also been shown to be unrelated to the metastasis of head and neck squamous cell carcinoma [40]. In addition, the overexpression of LCN2 is associated with radio-resistance and recurrence in NPC patients [9] Thus, the role of LCN2 in carcinogenesis and its underlying mechanism may depend on the various types of cancers.

We further identified that LCN2 negatively regulated the activation of several migration-associated signal proteins, including src, c-raf, MEK, ERK and p38. Notably, we found that the LCN2 negatively regulated MET expression levels. An abnormal expression and activation of MET has been shown to promote the occurrence and progression of many cancer types [41]. Indeed, a high MET protein expression level correlates with poorer survival in late-stage NPC [27]. The expression of MET in NPC tissues from patients with lymph node metastasis was significantly higher than that in NPC tissues from NPC patients without lymph node metastasis [42]. It is currently known to affect the abnormal expression of MET and could promote the development and progression of NPC. Further research may explore how LCN2 affects MET expression during NPC metastasis, such as through transcriptional regulation or post-transcriptional modification.

Until now, many studies have demonstrated that several signal molecules may participate in the invasion and metastasis of NPC. For example, Capn4 promotes the invasion and metastasis of NPC [43]. Elevated serum c-Src levels are associated with a poor prognosis in NPC patients [44]. The expression of YBX3 is positively correlated with NPC metastasis [45]. An abnormal expression of COX-2 is associated with recurrence and a poor prognosis in NPC patients [46]. Additionally, the downregulation of TNFAIP3 is associated with distant metastasis and a worse patient prognosis [47]. In the present study, we found that a low LCN2 level increased the expression of MET, resulting in an increase in the invasion and metastasis ability of NPC cells. Future studies may explore the interaction of these molecules in clinical NPC samples and establish a novel prognostic panel for the early detection of metastatic NPC.

The current study still had some limitations. Here, we used a bioinformatic analysis of a nasopharyngeal carcinoma database to support the finding that LCN2 reduced MET levels to inhibit nasopharyngeal cancer cell migration. In the future, human nasopharyngeal carcinoma tissues or animal models could be used to further validate our findings.

## 5. Conclusions

In summary, we found that LCN2 negatively controlled cell invasion and metastasis by increasing the expression level of MET in NPC cells. To our knowledge, this is the first study to show that LCN2 inhibits MET expression, resulting in the prevention of NPC metastasis through a novel molecular mechanism. Our study indicated that LCN2 may be a novel target for monitoring and treating the metastatic ability of patients with NPC. The results from our study may help to develop new biomarker panels and provide therapeutic targets for metastatic NPC.

## Data Availability

The datasets generated for this study are available on request to the corresponding authors.

## Supplementary Materials

The following are available online at https://www.mdpi.com/article/10.3390/cancers14225707/s1, File S1: The whole Western blot figures.
