# Supplementary material for: Lipocalin 2 Reduces MET Levels by Inhibiting MEK/ERK Signaling to Inhibit Nasopharyngeal Carcinoma Cell Migration
Source: Cancers (Basel). 2022 Nov 21;14(22):5707. doi: 10.3390/cancers14225707 (PMC9688489; doi:10.3390/cancers14225707)
Supplement: Supplementary file 1 [file cancers-14-05707-s001.zip › cancers-2044024-supplementary file.pdf]

Supplementary File

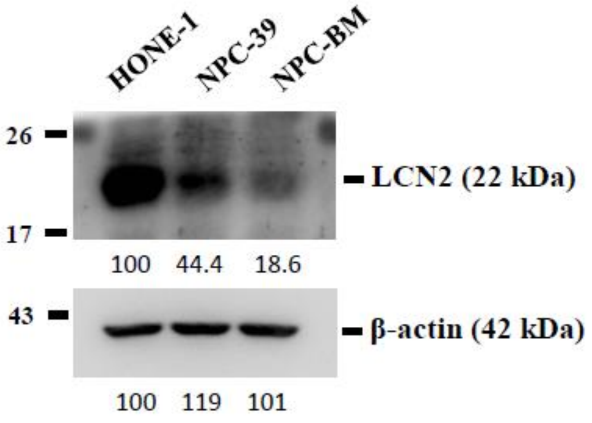

Figure S1. Refer to Figure 1B in the main text.

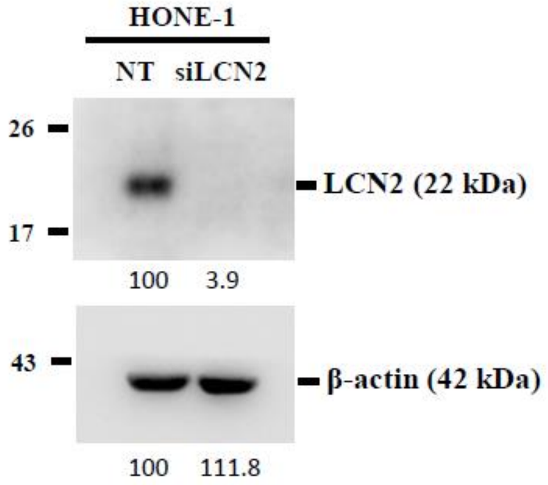

Figure S2. Refer to Figure 1D in the main text.

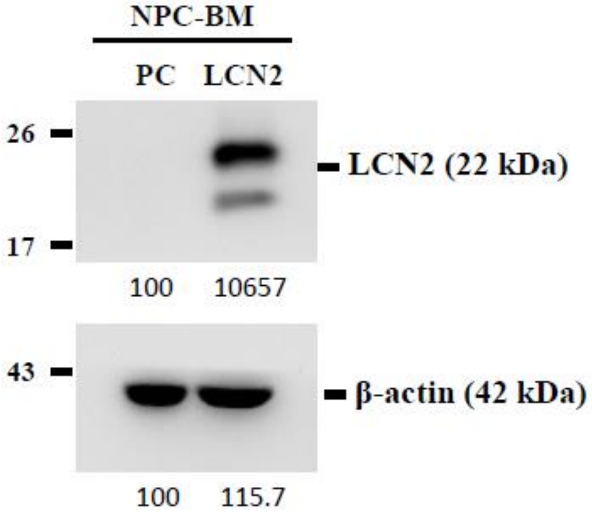

Figure S3. Refer to Figure 1E in the main text.

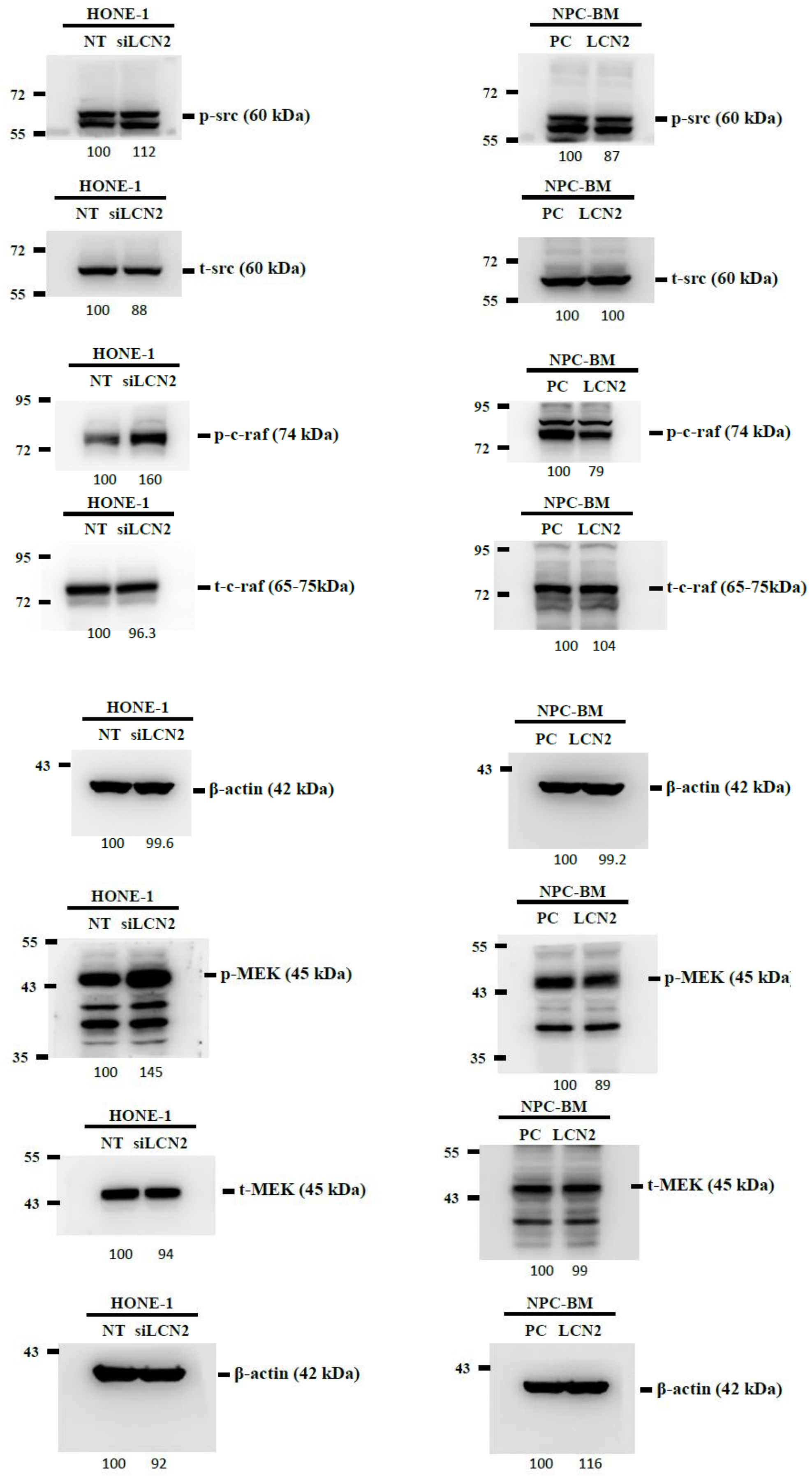

Figure S4. Refer to Figure 3A in the main text.

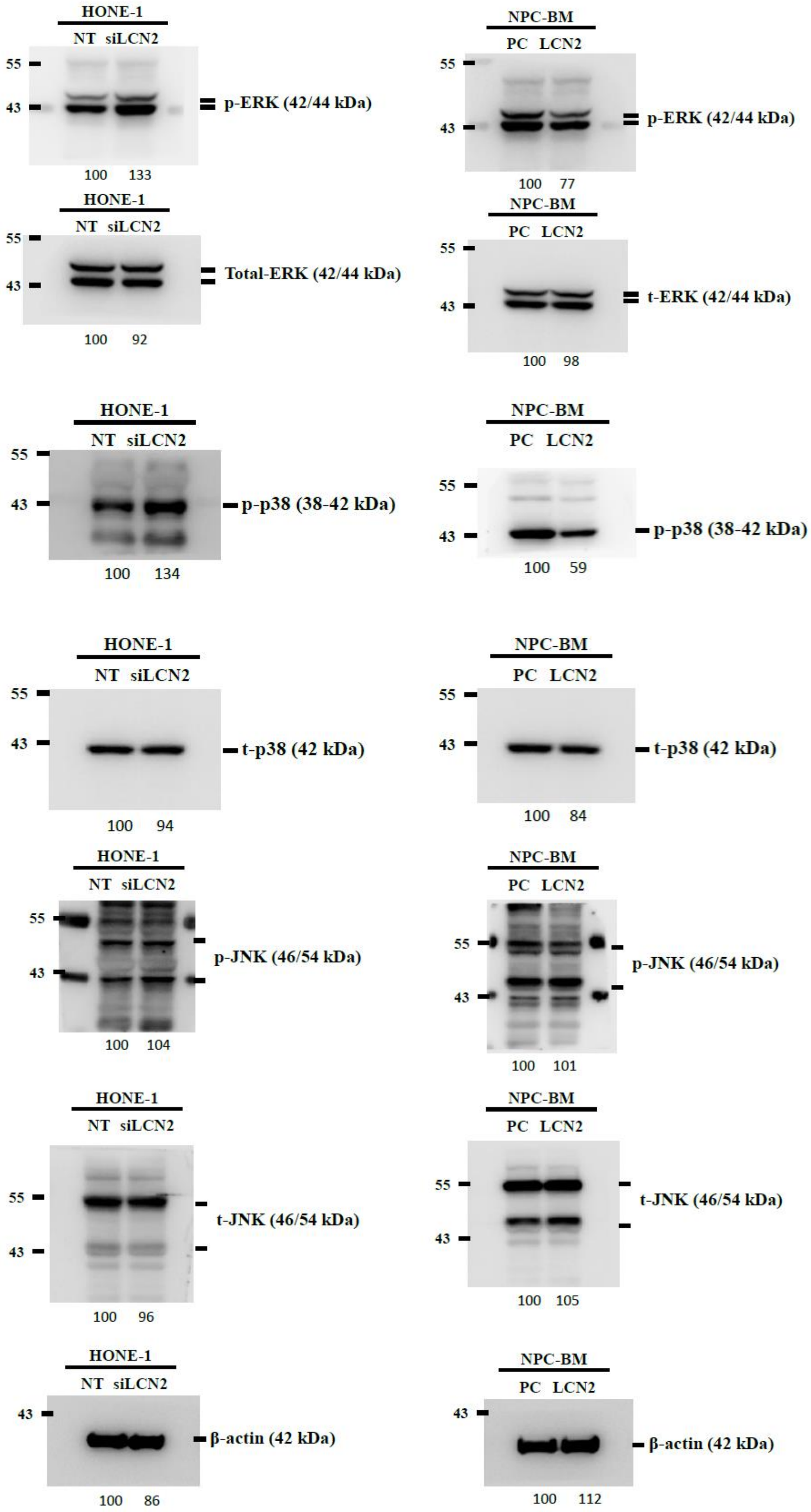

Figure S5. Refer to Figure 3B in the main text.

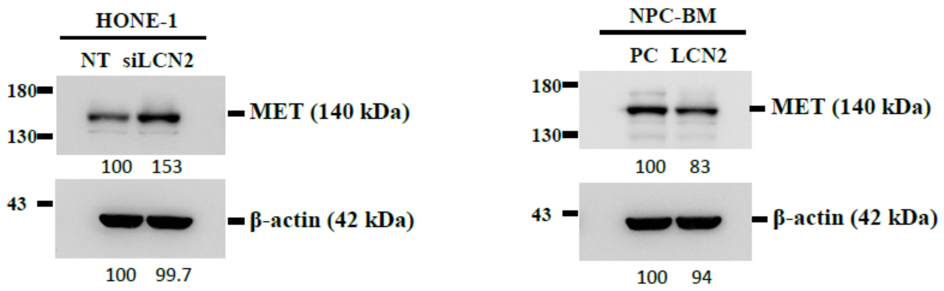

Figure S6. Refer to Figure 4D in the main text.

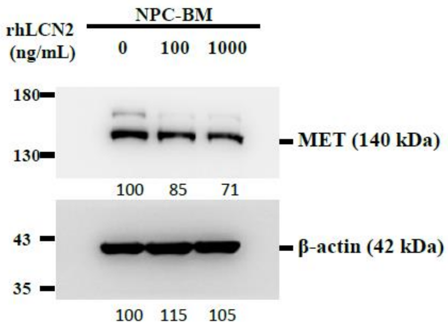

Figure S7. Refer to Figure 4G in the main text.

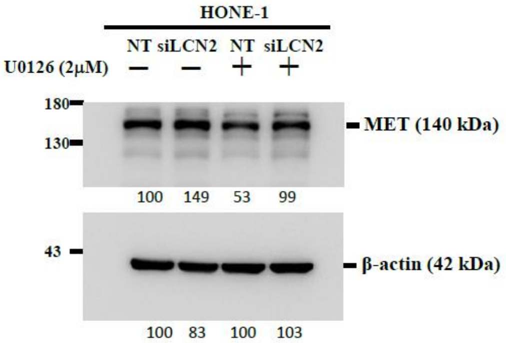

Figure S8. Refer to Figure 4H in the main text.

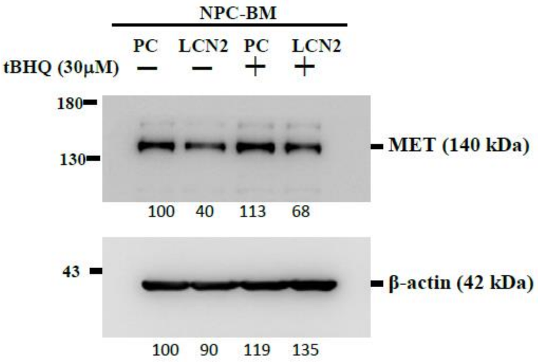

Figure S9. Refer to Figure 4I in the main text.

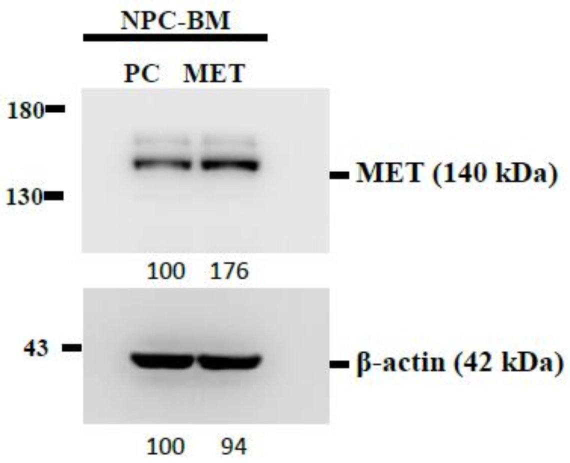

Figure S10. Refer to Figure 5A in the main text.

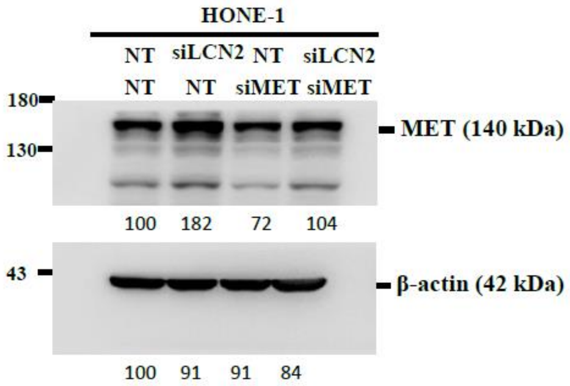

Figure S11. Refer to Figure 5C in the main text.
